# The Effects of Exercise Training on Cardiopulmonary Exercise Testing and Cardiac Biomarkers in Adult Patients with Hypoplastic Left Heart Syndrome and Fontan Circulation

**DOI:** 10.3390/jcdd9060171

**Published:** 2022-05-27

**Authors:** Marco Alfonso Perrone, Elettra Pomiato, Rosalinda Palmieri, Giulia Di Già, Fiorella Piemonte, Ottavia Porzio, Maria Giulia Gagliardi

**Affiliations:** 1 Department of Pediatric Cardiology and Cardiac Surgery, Bambino Gesù Children’s Hospital IRCCS, 00165 Rome, Italy; elettra.pomiato@opbg.net (E.P.); rosalinda.palmieri@opbg.net (R.P.); giulia.digia@opbg.net (G.D.G.); mgiulia.gagliardi@opbg.net (M.G.G.); 2 Unit of Neuromuscular and Neurodegenerative Diseases, Bambino Gesù Children’s Hospital IRCCS, 00165 Rome, Italy; fiorella.piemonte@opbg.net; 3 Unit of Clinical Pathology, Bambino Gesù Children’s Hospital IRCCS, 00165 Rome, Italy; ottavia.porzio@opbg.net

**Keywords:** aerobic exercise training, HLHS, exercise tolerance, cardiac biomarkers, cardiopulmonary exercise testing

## Abstract

Background: Several studies have shown that adult patients with Hypoplastic Left Heart Syndrome (HLHS) and Fontan circulation have a reduced exercise tolerance that affects daily life. Recent studies have investigated the effects of aerobic exercise training in patients with univentricular heart; however, this research topic is still poorly studied. The aim of this study was to evaluate the effects of an aerobic exercise training program on cardiopulmonary exercise testing parameters and cardiac biomarkers in patients with HLHS. Methods: We enrolled 12 patients with a mean age of 24 ± 2.5 years (range 22–27 years), 50% male, with HLHS at Bambino Gesù Children’s Hospital IRCCS. All patients underwent a cardiopulmonary test and blood sampling before (T0) and after (T1) a 4-week aerobic exercise program. Cardiac biomarkers hs-cTnT, NT-proBNP, ST2, GDF-15 were studied. Results: Data analysis demonstrated an increase in cardiorespiratory performance after 4 weeks of aerobic exercise training activity. In particular, the data showed a significant improvement in test duration (*p* < 0.05), heart rate at rest (*p* < 0.05), heart rate recovery 1 min (*p* < 0.05), VO2 max (*p* < 0.01) and oxygen uptake efficiency slope (*p* < 0.05). At the same time, the data showed a significant reduction in NT-proBNP and ST2 values (*p* < 0.01 and *p* < 0.05, respectively) and a significant increase in GDF-15 (*p* < 0.01). No significant changes were found between the hs-cTnT values. Conclusions: Our study demonstrated the 4-week efficacy of an aerobic training program in improving cardiorespiratory performance and cardiac biomarker values in adult patients with HLHS and Fontan circulation. More studies with larger numbers of patients will be needed to confirm these data.

## 1. Introduction

Hypoplastic left heart syndrome (HLHS) describes a heterogeneous group of diseases characterized by the underdevelopment of the structures of the left side of the heart, which include: the mitral valve, the left ventricle, the aortic valve and the aortic vessel. The term potentially indicates any malformation that determines an underdevelopment of the left heart structures, from aortic stenosis and aortic coarctation to aortic and mitral atresia and hypoplasia of the ascending aorta [1]. HLHS occurs in 0.16–0.36 per 1.000 live births and accounts for 1.4–3.8% of congenital heart disease [2]. Despite the relatively low incidence, it is responsible for 23% of cardiac-related deaths that occur in the first week of life. Until a few decades ago, children with HLHS had an almost total mortality in the first weeks of life; today, thanks to clinical and surgical innovations, these children are subjected to three delicate cardiac surgeries that allow them to grow and even reach adult life [2].

After these surgeries, patients with HLHS live with unusual circulation, supported by a single right ventricle. However, these patients with a single right ventricle have reduced exercise tolerance and an earlier incidence of ventricular dysfunction compared to patients with a normal heart or single left ventricle, and often suffer even worse outcomes [3]. Several studies have shown that exercise training improves clinical condition and quality of life in patients with Fontan circulation. In particular, recent studies have shown that aerobic physical activity improves exercise tolerance and cardiorespiratory performance in patients with Fontan circulation [4,5]; however, almost all cases are made up of left univentricular hearts. Therefore, the effects of aerobic physical activity in patients with HLHS are still poorly studied to date. At the same time, it has been shown how physical activity can have positive effects on Fontan circulation, with feedback on the values of heart failure biomarkers [6]. However, even in this case, there are still little data on the effects of exercise on cardiac biomarkers in patients with HLHS. 

In consideration of the positive effects that aerobic physical activity demonstrated in patients with single left ventricle [4,5], we hypothesized that an exercise training program could also improve cardiorespiratory performance in patients with Fontan circulation and single right ventricle. In particular, among the parameters of the cardiopulmonary test, our objective was to investigate whether the training program significantly improved heart rate (HR) and oxygen uptake by evaluating VO2 and oxygen uptake efficiency slope (OUES) and/or reduced VE/VCO2 slope.

Therefore, the aim of this study was to evaluate the effects of an aerobic exercise program on cardiopulmonary exercise testing and cardiac biomarkers (hs-cTnT, NT-proBNP, ST2 and GDF-15) in adult patients with Hypoplastic Left Heart Syndrome (HLHS) and Fontan circulation.

## 2. Methods

### 2.1. Patient Enrollment

We enrolled 12 patients with a mean age of 24 ± 2.5 years (range 22–27 years), 50% male, with HLHS and Fontan circulation at the Division of Cardiology of Adults with Congenital Heart Disease at Bambino Gesù Children’s Hospital IRCCS. Exclusion criteria were NYHA class III-IV, severe valvulopathies, osteo-muscular diseases, recent infections, hemodynamic instability, and PMK/ICD implantation. The study was approved by the ethics committee of Bambino Gesù Children’s Hospital IRCCS (protocol code 2610) and all patients signed informed consent. The study was conducted in accordance with the Declaration of Helsinki.

### 2.2. Study Protocol

After enrollment, all patients performed a venous blood sample, a cardiological visit and a cardiopulmonary exercise testing (T0). Then, they started an aerobic training program for 4 weeks. At the end of the 4 weeks (T1), the same patients underwent repeated blood sampling, cardiological examination and cardiopulmonary testing. 

### 2.3. Aerobic Training Program

The aerobic training program was established by the Division of Cardiology of Adults with Congenital Heart Disease at the Bambino Gesù Pediatric Hospital IRCCS in accordance with the current guidelines of the European Society of Cardiology. (ESC) [7,8]. 

The aerobic training program included:

- Three sessions per week for 4 weeks.
- A total of 30 min of walking or running on a treadmill or exercise bike with an intensity equal to 70% of the heart rate peak reached in the cardiopulmonary exercise testing (CPET).
- A 5 min warm-up of the same exercise to 40–50% of the max heart rate (HR) reached at CPET, before the exercise session.
- Then, 5 min of the same exercise at 40–50% of the max HR reached at CPET, after the exercise session.

Each patient could choose the most applicable option and it always remained the same. During training at home, each patient was provided with a heart rate monitor (Gima Spa, Gessate, Italy) and HR could be monitored through the device connected to the hospital via telemedicine.

### 2.4. Cardiopulmonary Stress Testing

All patients performed a cardiopulmonary test and blood sampling before (T0) and after (T1) a 4-week aerobic exercise training program. A cardiopulmonary exercise test was performed at the Division of Cardiology of Adults with Congenital Heart Disease. Breath by breath data were obtained throughout the entire exercise and analyzed by Cosmed Omnia Quark CPET^®^ Software (COSMED Srl, Rome, Italy). Electrocardiogram was continuously monitored at rest and during exercise until the fifth minute of recovery (Mortara^®^ XScribe™, Mortara Instrument Inc., Milwaukee, WI, USA). Oxygen saturation was monitored continuously from the beginning of exercise until the first minute of recovery. Blood pressure was measured at rest, every three minutes during exercise, at peak of exercise and thereafter until the fifth minute of recovery. The following parameters were evaluated for the study: test duration, resting and maximal systolic pressure (PAS), resting and maximal diastolic pressure (PAD), resting and maximal heart rate (HR), heart rate recovery at 1 min (HHR 1 min), VO2 max, VO2 predicted according to Wasserman [9], oxygen saturation (SpO2) at rest and max, VE/VCO2 slope and oxygen uptake efficiency slope (OUES).

### 2.5. Blood Sampling and Cardiac Biomarkers

All patients underwent blood vein sampling. After collection, the samples were placed on ice and analyzed at the Department of Laboratory Medicine and the Department of Neuroscience. For the study, high sensitivity troponin T (ng/L) and NT-proBNP (pg/mL) were evaluated with immunoassays using Roche^®^ platform (Roche Diagnostics, Mannheim, Germany), according to the manufacturer’s instructions. ST2 (ng/mL) was measured with the ELISA kit Critical Diagnostics Presage^®^ ST2 (Critical Diagnostics, San Diego, CA, USA), according to the manufacturer’s instructions. GDF-15 (pg/mL) was measured with the ELISA kit BioVendor R&D^®^ (BioVendor Research and Diagnostic Products, Brno, Czech Republic), according to the manufacturer’s instructions.

### 2.6. Statistical Analysis

Statistical analysis was performed using SPSS 21.0 software (IBM Corporation, Armonk, NY, USA). The Shapiro–Wilk normality test was used to assess normal distribution. Differences between the two groups were obtained using Student’s *t*-test. Values are indicated with mean ± standard deviation. Values of *p* < 0.05 were considered significant.

## 3. Results

All patients completed the study protocol. There were no major adverse cardiac events or arrhythmic episodes during the study. The cardiopulmonary test results are shown in Table 1.

Statistical analysis showed a significant increase in CPET duration after aerobic training (*p* < 0.05) and a reduction at T1 in HR at rest (*p* < 0.05). The data also showed a significant reduction in HHR 1 min after aerobic (*p* < 0.05). Regarding the ventilatory parameters, the data analysis showed a significant increase in VO2 max (*p* <0.01), VO2 predicted according to Wasserman (*p* < 0.05) and OUES (*p* < 0.05), while there were no significant differences between the other parameters evaluated (Table 1).

Regarding the laboratory parameters, the results of cardiac biomarkers are shown in Table 2.

Statistical analysis did not show a significant difference between T0 and T1 in hs-cTnT values (*p* > 0.05). We observed a significant reduction in NT-proBNP (*p* < 0.01) and ST2 (*p* < 0.05) values between T0 and T1. Finally, the data showed a significant increase in GDF-15 (*p* < 0.01) values after the exercise training program (Table 2).

## 4. Discussion

The aim of this study was to evaluate the effects of an aerobic exercise training program on cardiopulmonary exercise testing parameters and heart failure biomarkers in a group of adult patients with HLHS and Fontan circulation. Physical activity is globally recognized as an important prevention factor for cardiovascular disease and several experimental and clinical studies have shown that regular physical activity is associated with a reduction in cardiovascular risk and the death rate from heart disease [6,7,10]. Recent studies have shown that exercise programs increased exercise tolerance and cardiorespiratory performance, even in patients with such extraordinary circulation as Fontan circulation [11,12]. However, Scheffers et al. [13] demonstrated in a systematic review of the literature that studies are often conducted in groups including both pediatric and adult patients, and that the majority of enrolled patients have a single left ventricle. Even in the few studies that take into consideration only adult patients, almost all patients had a single left ventricle. The physiology of the ventricle in Fontan circulation dramatically changes the patient outcome [3,12]. Therefore, the type and regimen of physical activity should be adapted to the age, capacity and morbidity of each patient, possibly in homogeneous groups for age and underlying disease. 

Our data demonstrated that a 4-week aerobic training program significantly increases CPET duration (*p* < 0.05) in patients with HLHS. At the same time, the effects of training are also visible with a significant reduction at T1 in HR at rest (*p* < 0.05) and in HHR 1 min (*p* < 0.05). In agreement with previous studies [11,12,13], our data also showed a significant increase in VO2 max (*p* < 0.01) and in the % of predicted VO2 according to Wasserman (*p* < 0.05). Furthermore, we found a significant increase in OUES values at T1 (*p* < 0.05). To date, no studies have evaluated OUES values before and after a physical activity program in patients with HLHS. Therefore, data demonstrate an improvement in exercise tolerance and cardiorespiratory performance after an exercise training program. Several studies have already shown that physical activity improves the clinical condition of patients with a single ventricle. More specifically, some studies have shown that the skeletal muscle pump of the lower limbs improves the flow parameters of Fontan circulation during exercise [14,15]. Furthermore, we observed an improvement, albeit not significant, also in the VE/VCO2 slope. This parameter reflects the increase in ventilation in response to CO2 production, and thus shows increased ventilatory drive. In fact, several studies have shown that an increase in VE/VCO2 slope correlates with ventricular dysfunction and mortality in adult patients with heart failure or non-cyanotic congenital heart disease [16,17]. Instead, we found no significant differences in blood pressure and saturation parameters. 

Regarding the biomarkers of heart failure, several studies have shown that their increase is correlated with the incidence of re-hospitalization and with the mortality rate [18,19,20,21,22,23,24]. In particular, an increase in hs-cTnT, NT-proBNP, ST2 and GDF-15 is correlated with high mortality in both patients with biventricular and univentricular heart [21,22,23,25,26,27], while a reduction in these cardiac biomarkers, depending on drug therapy or other therapeutic approaches, is correlated with a reduction in mortality and an improvement in the clinical conditions of patients [18,28,29,30,31]. The analysis of our data showed no significant variation in the values of hs-cTnT (*p* > 0.05). Troponin is a marker of myocardial injury, and an increase in heart failure patients correlates with a worse outcome [28,32,33]. After our aerobic training program, however, the data showed a significant reduction in NT-proBNP (*p* < 0.01) and ST2 (*p* < 0.05). A reduction in these biomarkers correlates with a reduction in mortality and an improvement in the clinical condition of patients [22,23,29,34,35,36,37]. However, the most interesting data that emerged from the statistical analysis is the significant increase between T0 and T1 in the levels of GDF-15. Several studies have shown that an increase in GDF-15 correlates with worsening clinical conditions in patients with heart failure or Fontan circulation [27,37,38,39], but studies report usual values above the 99th percentile upper reference limit (URL) [39,40,41]. In our study, all GDF-15 values were below URL (1000 pg/mL). This increase was also observed by Kleinert et al. [42] and is explained by the fact that GDF-15, in addition to cardiac action, is a marker involved in the response to stress and in the modulation of metabolism. In mouse skeletal muscle, GDF-15 expression increases markedly in response to mitochondrial stress [42,43], which can lead to increased plasma levels of GDF-15. In humans, circulating levels of GDF-15 are elevated in patients with muscle atrophy [43] and in patients with mitochondrial myopathy [44]. These data suggest that in response to a stress stimulus, skeletal muscle could release GDF-15 into the circulation. Therefore, the increase in GDF-15 that we found at T1 could be due to an increase in the physiological muscle stress due to the training program, with beneficial effects on the regulation of metabolism. This is the first study to evaluate the levels of cardiac biomarkers after an aerobic training program in patients with HLHS. 

### Study Limitations

A limitation of our study is the number of patients enrolled. This is due to the peculiarity of the studied population of adult patients with HLHS and Fontan circulation and the number is consistent with that of other similar studies [6,45]. Another limitation of the study is that it did not consider a control group. Several studies have shown that exercise training improves exercise tolerance in patients with Fontan circulation and single left ventricle [12,13]. Assuming, based on the very few existing studies [6,45], a similar effect also in patients with single right ventricle, we decided not to deprive a group of control patients of this possibility.

## 5. Conclusions

The data from our study showed that a 4-week program of aerobic exercise training improved cardiopulmonary test parameters with an increase in exercise tolerance and an improvement in cardiorespiratory performance. At the same time, the 4-week aerobic training program induced positive effects on heart failure biomarker values. More studies with a larger number of patients will be needed to confirm these data and to better investigate this important research topic, in order to perhaps reach a real therapeutic prescription of physical exercise in these patients.

## Figures and Tables

**Table 1:** Comparison of cardiopulmonary test parameters between T0 and T1. ns = not significant.

	T0 (Mean ± SD)	T1 (Mean ± SD)	*p*-Value
Test duration (s)	631.5 ± 96.5	692.3 ± 75.3	*p* < 0.05
HR at rest (bpm)	89.6 ± 12.9	86.6 ± 11.8	*p* < 0.05
HR max (bpm)	140.6 ± 25.7	144.6 ± 28.5	ns
HHR 1 min (bpm)	−14.6 ± 7.5	−18.6 ± 6.5	*p* < 0.05
PAS at rest (mmHg)	115.0 ± 8.3	112.5 ± 4.1	ns
PAD at rest (mmHg)	65.8 ± 6.6	65.6 ± 4.9	ns
PAS max (mmHg)	148.3 ± 7.5	146.6 ±15.0	ns
PAD max (mmHg)	66.6 ± 5.1	68.3 ± 7.5	ns
VO2 max (mL/kg/min)	21.5 ± 3.0	23.1 ± 2.3	*p* < 0.01
VO2 predicted according to Wasserman (%)	54.5 ± 11.8	58.5 ± 12.1	*p* < 0.05
SpO2 base (%)	94.8 ± 2.0	96.4 ± 1.9	ns
SpO2 max (%)	93.8 ± 1.8	94.6 ± 2.9	ns
VE/VCO2 slope	34.3 ± 8.9	33.8 ± 8.5	ns
OUES (mL/10logL)	1620.1 ± 177.0	1746.0 ± 240.7	*p* < 0.05

**Table 2:** Comparison of cardiac biomarker values between T0 and T1. ns = not significant.

	T0 (Mean ± SD)	T1 (Mean ± SD)	*p*-Value
Hs-cTnT (ng/L)	4.2 ± 0.8	3.1 ± 0.4	ns
NT-proBNP (pg/mL)	96.3 ± 66.7	62.5 ± 46.1	*p* < 0.01
ST2 (ng/mL)	23.6 ± 8.8	20.4 ± 7.8	*p* < 0.05
GDF-15 (pg/mL)	441.0 ± 94.2	590.1 ± 126.8	*p* < 0.01

## Data Availability

The data presented in this study are available on request from the corresponding author.
